# Are more otolaryngologists enough to improve surgical access? Geographic distribution and health system barriers in Brazil

**DOI:** 10.1016/j.bjorl.2026.101882

**Published:** 2026-09-02

**Authors:** Lucas Sousa Salgado, Sofia Fontes de Oliva, Rebecca Gallardo Franco de Andrade, Vagner Antonio Rodrigues Silva, Arthur Menino Castilho

**Affiliations:** Universidade Estadual de Campinas (UNICAMP), Department of Otorhinolaryngology and Head and Neck Surgery, Campinas, SP, Brazil

**Keywords:** Otolaryngology, Health services accessibility, Delivery of health care, Global health

## Abstract

**Objective:**

To examine the geographic distribution of otolaryngologists in Brazil and evaluate the association between specialist density and otolaryngologic surgical provision within the public health system between 2008 and 2024.

**Methods:**

This nationwide longitudinal study used data from the Hospital Information System of the Brazilian Unified Health System (SIH/SUS), combined with demographic and economic indicators obtained from the Brazilian Institute of Geography and Statistics. Otolaryngologist workforce density was defined as the number of practicing specialists per 100,000 inhabitants in each state. Surgical rates, procedure costs, and surgical productivity were analyzed by state and geographic region. Inequality in workforce distribution was assessed using the coefficient of variation and the Gini index. States were further grouped through k-means cluster analysis based on workforce density, surgical rates, and gross domestic product per capita. Mixed-effects regression models were applied to examine the association between specialist density and surgical provision over time, adjusting for economic indicators.

**Results:**

During the study period, 730,757 otolaryngologic procedures were performed within the Brazilian public health system. The national density of otolaryngologists increased from 2.41 to 3.97 specialists per 100,000 inhabitants between 2011 and 2024. Workforce distribution remained uneven across the country, with higher concentrations observed in the South and Southeast and substantially lower densities in the North and Northeast. Statistical modelling identified a significant association between specialist density and surgical rates (p < 0.001). However, the estimated number of procedures corresponding to each additional specialist decreased over time, from 11.6 surgeries in 2011 to 2.8 in 2024. Measures of inequality showed a reduction during the study period, with the Gini index declining from 0.33 to 0.27, although differences between regions persisted. Procedure costs varied geographically, with higher mean expenditures reported in the South and Southeast.

**Conclusion:**

The expansion of the otolaryngology workforce in Brazil was accompanied by comparatively smaller increases in surgical provision across regions. This pattern may reflect structural limitations within the public health system that extend beyond specialist availability. The findings suggest that improvements in surgical access may depend on broader system capacity.

**Level of evidence:**

3 – Ecological longitudinal (cross-sectional time-series) study.

## Introduction

Otolaryngology is a core surgical specialty that plays a crucial role in population health by addressing diseases that impair communication, breathing, and hearing. From chronic ear disease in children to head and neck cancer in adults, these conditions impose a high burden of disability and economic loss.^1^ Under universal health coverage, access to timely and safe otolaryngologic care is therefore a key determinant of social well-being and equity.^2^ In Brazil, where 76% of the population depends exclusively on the unified public health system (SUS), the equitable delivery of surgical care represents one of the greatest challenges of the public health agenda.^3^ Recognizing this, the Ministry of Health recently launched the “Now There Are Specialists” programme to expand the number of specialists in priority fields, including otolaryngology. The programme aims to correct workforce imbalances and strengthen capacity to meet surgical demand, but whether workforce growth alone translates into greater surgical provision remains uncertain.

This national debate aligns with the broader global movement for surgical equity, galvanized by the 2015 Lancet Commission on Global Surgery (LCoGS), which established that access to safe, timely, and affordable surgery is an essential component of universal health coverage.^4^ Surgical conditions account for nearly one-third of the global burden of disease,^5^ and workforce distribution has emerged as a key determinant of access, particularly in Low-Income and Middle-Income Countries (LMICs), where rural and underserved populations face persistent barriers to specialized care.^6^, ^7^, ^8^, ^9^ Within this framework, otolaryngology occupies a strategic position: it bridges essential, emergency, and elective procedures, serving both pediatric and adult populations.

Despite the growing global interest in surgical workforce distribution, no previous research has specifically evaluated the geographic distribution of otolaryngologists in Brazil or its implications for surgical care delivery. This knowledge gap is critical, given Brazil’s continental dimensions, marked socioeconomic disparities, and the SUS mandate to ensure universal access, as it represents one of the largest public health systems in the world.^3^ Understanding how the spatial distribution of otolaryngologists correlates with surgical productivity and access is essential to inform targeted workforce policies. Such evidence can guide strategic investments that not only expand specialist availability, but also translate workforce expansion into measurable improvements in population health. Therefore, the present study aims to analyze the geographic distribution of otolaryngologists across Brazil and assess its association with the provision of surgical care within the public health system.

## Methods

### Study design and data sources

This ecological longitudinal study examined the association between the geographic distribution of otolaryngologists and the provision of otolaryngologic surgery in Brazil’s public health system from 2008 to 2024. Earlier Hospital Admission Authorizations (HAA) data (1992–2007) were excluded because they were highly incomplete, unevenly distributed across states, affected by substantial underreporting, and lack consistent coverage of key otolaryngologic surgical procedure codes, limiting their suitably for national or regional analyses and for reliable temporal comparisons. Data on surgical volume were obtained from the Hospital Information System of the Unified Health System (SIH/SUS).^10^ Surgical procedures were identified based on approved HAA, which represent the official administrative and reimbursement records for all inpatient procedures performed within the public health system. Only approved HAA records were included, ensuring that all procedures analyzed corresponded to completed and reimbursed hospitalizations.

Each HAA record is linked to a specific SIH/SUS procedure code and its official procedure name, which are displayed in the query interface. For this study, we selected all SIH/SUS procedure codes corresponding to otolaryngologic surgeries available in the system, grouped according to surgical category and clinical indication, to ensure comprehensive capture of inpatient surgical activity within the specialty.

Procedures with a substantial oncologic component were excluded because SIH/SUS coding does not reliably distinguish benign from malignant indications. In addition, oncologic head and neck surgery represents a distinct clinical domain with substantially different complexity, costs, and care pathways. Highly specialized implant-based procedures, including cochlear implantation and bone-anchored hearing systems, were also excluded because they rely on specific funding mechanisms, limited-service availability, and centralized referral networks. These exclusions were made to preserve internal consistency and ensure a robust assessment of routine otolaryngologic surgical provision within the public health system.

The following otolaryngologic surgical procedures and corresponding SIH/SUS codes were included. Adenotonsillectomy was identified using the code 0404010032 (adenotonsillectomy, “amigdalectomia com adenoidectomia”). Mastoidectomy procedures comprised subtotal mastoidectomy (code 0404010229, “mastoidectomia subtotal”) and radical mastoidectomy (code 0404010210, “mastoidectomia radical”).

Septoplasty included procedures for septal deviation correction (codes 0404010482 and 0404020330, “septoplastia para correção de desvio”), reconstructive non-aesthetic septoplasty (codes 0404010520 and 0414010230, “septoplastia reparadora não estética”), and septoplasty performed in patients with craniofacial anomalies (code 0404030173, “septoplastia em paciente com anomalia cranio e bucomaxilofacial”).

Sinusotomy procedures were identified through bilateral sinusotomy (code 0404010326, “sinusotomia bilateral”), unilateral maxillary sinusotomy (code 0414020324, “sinusotomia maxilar unilateral”), sphenoidal sinusotomy (code 0404010334, “sinusotomia esfenoidal”), and transmaxillary sinusotomy (codes 0404010512 and 0404030181, “sinusotomia transmaxilar”). In the SIH/SUS coding system, frontal and ethmoidal sinus procedures were not available as standalone procedure codes; instead, these approaches are incorporated within the broader sinusotomy categories, depending on the surgical access route and the classification recorded in the approved HAA.

Finally, tympanoplasty included unilateral or bilateral tympanoplasty (code 0404010350, “timpanoplastia uni/bilateral”) and tympanoplasty performed in patients with craniofacial anomalies, unilateral or bilateral (code 0404030190, “timpanoplastia em paciente com anomalia cranio e bucomaxilofacial”).

Population size and Gross Domestic Product (GDP) per capita were retrieved from the Brazilian Institute of Geography and Statistics (IBGE).^11^ The number of practicing otolaryngologists was drawn from the National Registry of Health Establishments. Although surgical data were available from 2008 onward, analyses involving otolaryngologist density were restricted to 2011 onward, when nationwide standardized workforce data became consistently available.

### Variables

The primary outcome was the annual rate of selected otolaryngologic procedures per 100,000 inhabitants, aggregated at the state level. Procedures were selected based on their frequency and representativeness of routine otolaryngologic surgical practice, including adenotonsillectomy, mastoidectomy, septoplasty, sinusotomy, and tympanoplasty. The main exposure was otolaryngologist density, defined as the number of registered specialists per 100,000 inhabitants, with GDP per capita (in USD) and calendar year as covariates.

### Statistical analysis

Descriptive analyses were conducted for all variables by federative unit and geographic region, including measures of central tendency and dispersion. Inequality in the distribution of otolaryngologists across states over time was assessed using the Coefficient of Variation (CV) and the Gini index. Linear associations between GDP per capita, otolaryngologist density, and surgical rate per capita were examined using Pearson’s correlation coefficient. State-level heterogeneity was further explored through κ-means cluster analysis, incorporating otolaryngologist density, surgical rate per capita, and GDP per capita. Cluster structure was visualized with scatter plots, and differences between clusters were assessed using analysis of variance (ANOVA).

State-level heterogeneity was explored using k-means cluster analysis based on otolaryngologist density per 100,000 inhabitants, otolaryngologic surgical rates, and GDP per capita. Prior to clustering, all variables were standardized (z-scores) to ensure comparability across states. The number of clusters was set to κ = 4, defined a priori to balance interpretability and stability of the clustering solution. States were assigned to one of the four clusters based on the nearest centroid, minimizing within-cluster sum of squares. Cluster structure was visualized using scatter plots, and between-cluster differences in the input variables were subsequently evaluated using ANOVA.

To evaluate the relationship between specialist density and surgical output over time, we fitted a mixed-effects linear regression model with random intercepts for states, adjusting for GDP per capita. The model included interaction terms between otolaryngologist density and year, to estimate temporal variation in the strength of this association. The dependent variable was the surgical rate per capita, and fixed effects included otolaryngologist density, GDP per capita, and year. Clusters containing a single observational unit were retained for descriptive and graphical purposes but were excluded from inferential statistical comparisons due to the impossibility of estimating within-cluster variability. Statistical significance was defined as p < 0·05. All analyses were performed in R (version 4.4.2).

### Ethical considerations

This study used publicly available, de-identified data and was therefore exempt from institutional ethics committee review, in accordance with Brazilian National Health Council Resolution 510/2016.

## Results

### Otolaryngologist distribution and availability

**Fig. 1:**
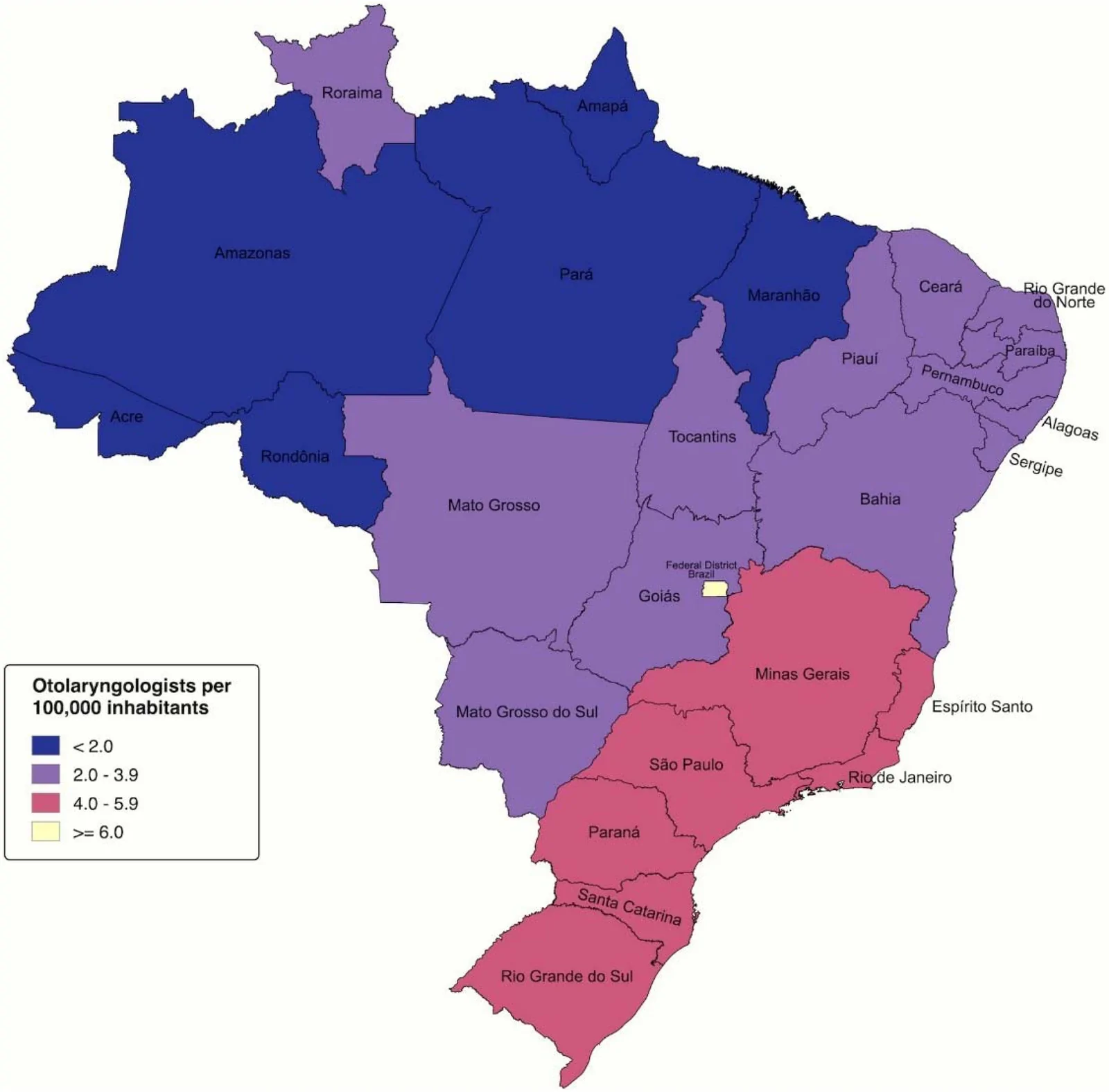
Density of otolaryngologists per 100,000 inhabitants in Brazil, by state (2024). Higher densities are observed in the Southeast and South, with markedly lower values in the North and Northeast.

Between 2008 and 2024, a total of 730.757 otolaryngologic surgical procedures were recorded in the Brazilian public health system. Adenotonsillectomy represented the majority of procedures (508,545; 69.6%), followed by septoplasty (96,736; 13.2%), tympanoplasty (63,338; 8.7%), sinusotomy (32,275; 4.4%), and mastoidectomy (29,863; 4.1%). The geographic distribution of otolaryngologists was markedly heterogeneous across Brazil (Fig. 1). Nationally, the mean density of otolaryngologists increased from 2.41 per 100.000 inhabitants in 2011 to 3.97 per 100.000 in 2024, corresponding to a 64.7% increase during the study period. The Southeast consistently concentrated the largest share of specialists, with São Paulo reaching 5.92 per 100.000 in 2024, whereas the lowest values were observed in Amapá (1.25), Roraima (0.95), and Acre (1.14). Across regions, the Southeast maintained the highest density throughout the period (3.24 → 5.06), followed by the South (3.14 → 4.64) and Central-West (2.64 → 4.45), whereas the North (0.81 → 1.49) and Northeast (1.20 → 2.56) remained the lowest despite substantial relative gains.

Absolute variability between states increased over time, with the standard deviation of specialist density rising from 1.17 to 1.74. However, the Coefficient of Variation (CV) decreased from 0.613 in 2011 to 0.527 in 2024, and the Gini index declined from 0.327 to 0.267. This pattern indicates that, although the absolute spread in densities increased, the proportional disparity between states with the highest and lowest specialist densities became smaller over time.

### Surgical production and regional gradient

Throughout the study period, the South presented the highest surgical rates per capita, followed by the Southeast, whereas the North consistently showed the lowest values. In 2024, Rio Grande do Sul recorded 130.5 surgeries per 100,000 inhabitants compared with 19.8 per 100,000 in Amapá. GDP per capita followed a similar regional distribution, with the highest values observed in the South and Southeast and the lowest in the North and Northeast. In 2024, GDP per capita in São Paulo (USD 10,676) was more than three times higher than in Maranhão (USD 3,354). Surgical productivity, defined as procedures per specialist, declined substantially between 2011 and 2020, followed by partial recovery by 2024 (Table 1). Mean productivity decreased from 10.9 surgeries per otolaryngologist in 2011 to 2.9 in 2020, increasing to 7.0 in 2024. The South and Southeast remained above the national mean throughout the period. Paraná and Santa Catarina showed marked declines during the pandemic years followed by recovery. Smaller states such as Roraima displayed considerable year-to-year variability, whereas Acre and Paraíba showed notable increases in 2024. In contrast, Rio Grande do Norte maintained a declining trend.

**Table 1:** Annual number of otolaryngologic surgeries performed per practicing otolaryngologist in Brazil, calculated as the total number of surgeries divided by the number of practicing specialists in each year.

	2011	2013	2015	2018	2020	2024
State						
Acre	26.6	32.5	14.1	7.6	10.5	39.5
Alagoas	17.8	18.1	7.4	7.5	3.4	4.8
Amapá	1.4	0.3	2.6	4.2	1.6	3.4
Amazonas	2.5	2.6	2.4	0.8	1.3	1.5
Bahia	8.1	6.7	6.0	5.7	2.9	5.3
Ceará	6.6	4.4	3.6	2.8	1.5	5.5
Distrito Federal	5.1	4.9	2.3	2.2	1.1	4.2
Espírito Santo	18.0	6.5	8.9	6.2	2.1	6.7
Goiás	7.5	10.7	13.6	6.0	3.5	6.0
Maranhão	11.3	10.4	11.1	12.0	2.6	11.0
Mato Grosso	10.1	9.9	4.8	4.2	2.4	11.9
Mato Grosso do Sul	8.0	9.8	8.6	4.0	2.1	6.4
Minas Gerais	12.4	8.6	8.4	5.1	2.2	7.1
Pará	14.9	11.6	6.0	5.9	2.8	5.9
Paraíba	4.6	9.6	7.4	5.8	2.9	20.0
Paraná	19.8	13.2	9.7	10.9	3.5	18.1
Pernambuco	8.7	7.0	6.6	4.2	1.3	3.6
Piauí	4.4	4.1	5.9	5.3	1.2	2.2
Rio de Janeiro	6.1	5.3	4.6	4.7	2.2	3.5
Rio Grande do Norte	18.9	19.7	9.7	5.4	1.8	0.9
Rio Grande do Sul	7.5	6.8	6.7	6.6	3.2	5.8
Rondônia	6.8	4.9	6.4	5.8	1.3	2.5
Roraima	8.0	5.6	6.0	16.4	1.8	6.1
Santa Catarina	16.1	14.2	11.5	10.6	4.0	10.2
São Paulo	11.6	10.3	8.5	7.7	3.5	6.3
Sergipe	4.0	5.3	7.1	6.1	2.0	4.4
Tocantins	12.1	4.1	3.0	4.0	4.8	8.4
Region						
Central-West	7.0	8.2	7.5	4.0	2.2	6.2
Northeast	8.8	8.1	6.4	5.4	2.2	6.2
North	10.0	8.2	5.1	4.5	2.8	6.1
Southeast	11.1	8.9	7.8	6.6	3.0	6.0
South	13.9	10.8	8.8	9.1	3.5	11.6
Brazil	10.9	9.1	7.6	6.6	2.9	7.0

**Fig. 2:**
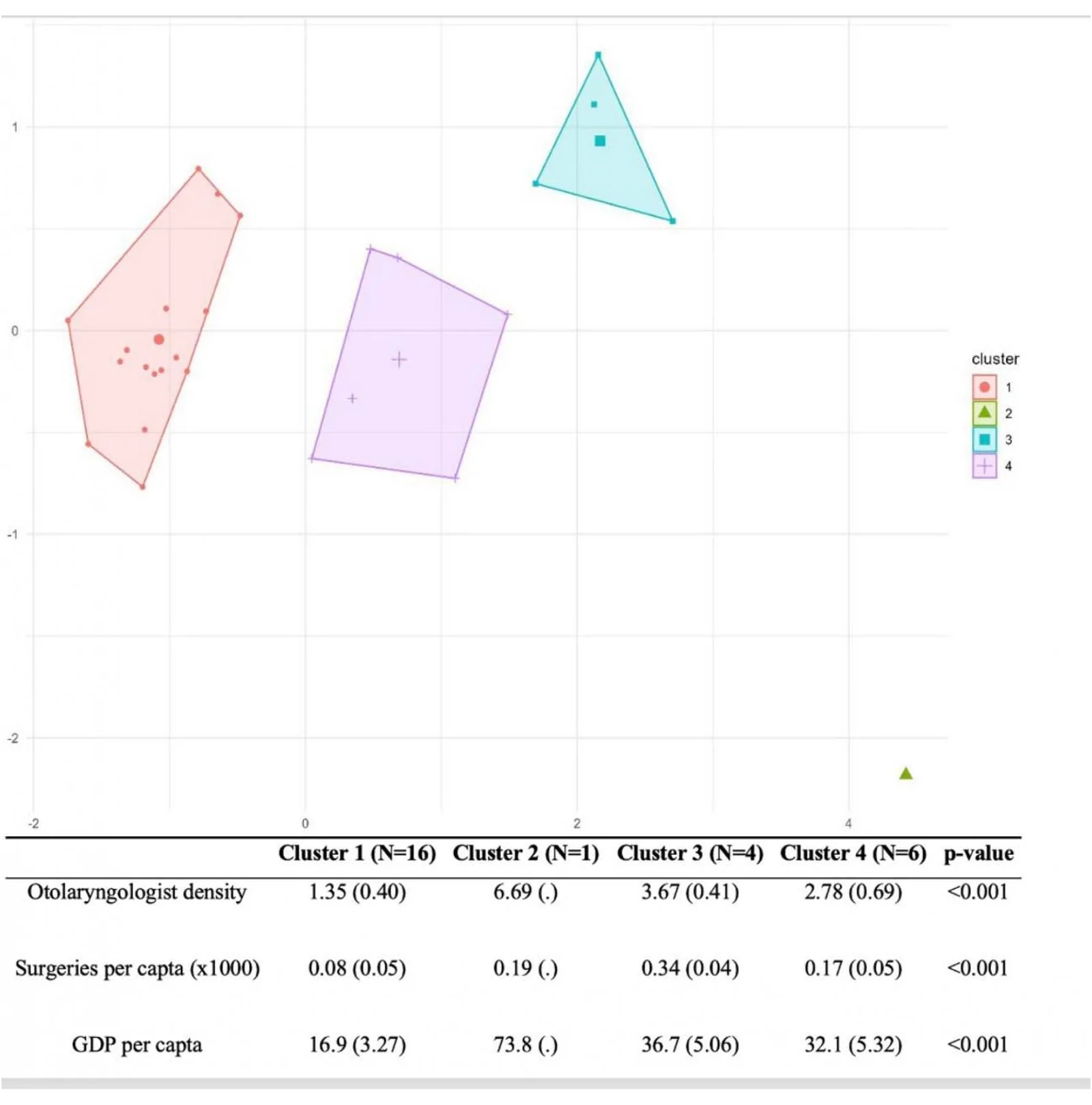
Cluster analysis of Brazilian states by otolaryngologist density, surgical rates per capita, and GDP per capita. Clusters: (1) Acre, Alagoas, Amapá, Amazonas, Bahia, Ceará, Maranhão, Pará, Paraíba, Pernambuco, Piauí, Rio Grande do Norte, Rondônia, Roraima, Sergipe, Tocantins; (2) Federal District; (3) Espírito Santo, Paraná, Santa Catarina, São Paulo; (4) Goiás, Mato Grosso, Mato Grosso do Sul, Minas Gerais, Rio de Janeiro, Rio Grande do Sul.

Cluster analysis identified four state profiles. Cluster 1 comprised mainly North and Northeast states and was characterized by the lowest values across all indicators. Cluster 2 included only the Federal District, an outlier with the highest specialist density and GDP per capita but moderate surgical rates, and was excluded from comparative statistical analyses. Cluster 3 included predominantly South and Southeast states with high specialist density and the highest surgical rates. Cluster 4 comprised states from the Central-West and parts of the Southeast with intermediate values. ANOVA confirmed statistically significant differences between clusters, with progressive increases in otolaryngologist density, surgical rates, and GDP per capita from Cluster 1 to Cluster 4 (Fig. 2).

### Costs and financing

Between 2008 and 2024, substantial regional differences were observed in the average public expenditure per otolaryngologic procedure (Table 2). Across the study period, the highest average costs were observed in the South and Southeast, whereas the lowest occurred in the North and Northeast. For mastoidectomy, the most complex otologic procedure included in the analysis, average expenditures reached USD 207.09 in the South in 2024 compared with USD 121.27 in the North. For routine procedures such as tonsillectomy and septoplasty, regional differences were smaller, although the same geographic pattern persisted.

**Table 2:** Mean public expenditure per otolaryngologic surgery (USD), by procedure type and region, Brazil, 2008–2024.

	State	2008	2009	2010	2011	2012	2013	2014	2015	2016	2017	2018	2019	2020	2021	2022	2023	2024
Adenoamigdalectomy	Central-West	53.27	62.18	64.91	66.91	72.55	98.73	115.64	135.27	126.18	121.82	86.91	96.55	80.73	90.0	89.82	101.27	119.09
	Northeast	53.45	65.82	63.64	65.27	65.27	88.18	75.09	72.36	69.82	76.36	68.91	68.0	61.64	65.64	67.27	75.27	122.36
	North	56.36	65.27	66.73	67.27	66.73	72.18	68.73	67.45	68.55	72.18	71.09	72.55	59.27	72.55	66.0	81.09	66.18
	Southeast	53.27	64.18	64.73	65.45	67.27	75.64	86.73	83.45	65.82	70.73	75.64	72.91	63.27	66.91	66.18	73.27	103.45
	South	51.64	62.0	62.91	63.27	64.91	77.64	82.18	81.09	63.27	70.36	74.91	72.73	60.18	71.64	67.27	82.55	125.82
	*Brazil*	52.91	63.64	64.0	64.91	66.55	79.27	86.18	85.82	71.27	75.64	74.91	73.45	63.45	69.64	68.18	78.55	113.64
Mastoidectomy	Central-West	76.91	124.36	133.64	132.18	132.18	176.91	202.36	227.45	153.27	144.91	169.45	156.0	177.09	140.0	152.91	176.73	168.91
	Northeast	91.45	133.27	131.27	140.73	129.82	130.55	122.36	130.0	146.0	131.09	127.45	152.73	125.64	190.55	165.27	158.73	159.27
	North	79.27	150.18	141.09	134.36	141.45	137.27	126.91	137.45	149.27	124.18	159.45	141.82	112.0	138.0	138.55	94.73	121.27
	Southeast	82.91	130.55	131.64	130.91	135.82	139.27	139.27	160.36	131.09	146.91	166.36	161.27	144.73	144.73	143.27	147.09	185.82
	South	89.27	130.73	137.09	160.91	138.91	154.0	155.45	145.09	155.09	137.45	146.73	146.91	147.09	156.0	151.27	170.73	207.09
	*Brazil*	84.73	131.64	132.91	136.73	135.27	142.73	143.82	158.55	139.45	142.18	157.64	156.55	144.73	150.91	147.09	152.73	178.91
Septoplasty	Central-West	43.45	74.73	76.73	64.55	47.09	55.45	62.91	68.18	59.82	57.64	56.18	59.45	87.82	89.09	64.91	72.55	89.82
	Northeast	46.91	79.09	77.09	72.55	45.09	47.82	48.36	44.91	47.27	46.0	48.0	52.91	67.64	41.27	44.91	55.45	65.45
	North	44.18	80.36	71.45	72.36	48.55	47.09	49.45	47.27	55.82	55.82	54.18	52.0	57.82	52.91	57.64	55.27	51.82
	Southeast	44.91	79.82	79.64	63.64	44.36	47.64	64.36	78.73	44.73	51.64	50.91	50.91	48.18	63.45	46.18	50.36	79.27
	South	46.91	78.55	78.91	68.73	44.91	51.27	59.27	57.09	46.18	46.18	48.0	49.45	42.91	50.55	49.09	47.82	71.82
	*Brazil*	45.27	79.27	78.91	65.09	44.73	48.91	62.36	72.0	46.55	50.73	50.55	51.45	51.27	60.73	49.27	52.0	77.09
Sinus Surgery	Central-West	38.36	69.82	80.36	100.55	83.09	114.55	95.27	102.18	104.18	87.64	82.18	91.82	119.09	152.36	136.91	130.73	127.27
	Northeast	49.45	73.64	70.55	70.73	84.36	75.82	75.09	78.36	79.82	90.91	85.82	74.55	106.91	92.55	103.64	110.73	125.45
	North	39.82	80.18	93.27	73.45	68.91	73.82	105.45	87.09	128.73	83.45	80.55	80.55	84.0	262.55	121.09	102.73	126.55
	Southeast	56.73	95.64	96.18	101.27	93.09	94.0	86.36	99.64	92.36	101.09	107.64	105.27	105.82	99.45	115.45	114.55	132.0
	South	60.0	74.18	85.82	119.64	104.55	140.18	103.64	94.18	109.45	101.64	96.91	100.91	84.73	117.09	117.82	136.91	157.45
	*Brazil*	54.36	87.82	90.0	98.0	92.0	98.0	88.55	96.18	96.0	98.73	100.73	99.45	102.73	108.18	116.36	118.55	134.0
Timpanoplasty	Central-West	75.45	112.73	112.91	112.73	120.91	135.45	162.36	175.82	124.73	128.36	119.27	123.27	138.73	140.18	126.18	144.36	133.27
	Northeast	80.0	112.55	113.64	115.82	113.09	129.64	117.27	114.91	135.64	125.64	120.91	119.09	110.55	112.18	116.36	116.18	149.64
	North	80.73	108.0	112.73	120.55	112.55	114.36	112.55	120.55	112.0	122.36	117.45	117.82	113.82	117.45	126.18	121.64	128.73
	Southeast	75.27	113.27	109.82	113.64	112.55	121.45	137.09	143.64	114.91	127.45	139.09	132.73	114.36	136.18	115.27	121.45	167.64
	South	77.09	110.91	114.36	110.55	113.64	120.0	126.55	122.73	113.45	113.27	126.18	114.18	109.45	169.45	115.64	114.18	173.45
	*Brazil*	76.36	112.55	111.45	113.45	113.27	123.82	132.36	135.45	119.64	124.0	131.45	125.09	114.0	139.09	116.91	121.64	161.27

### Multivariable analysis and determinants of surgical provision

**Fig. 3:**
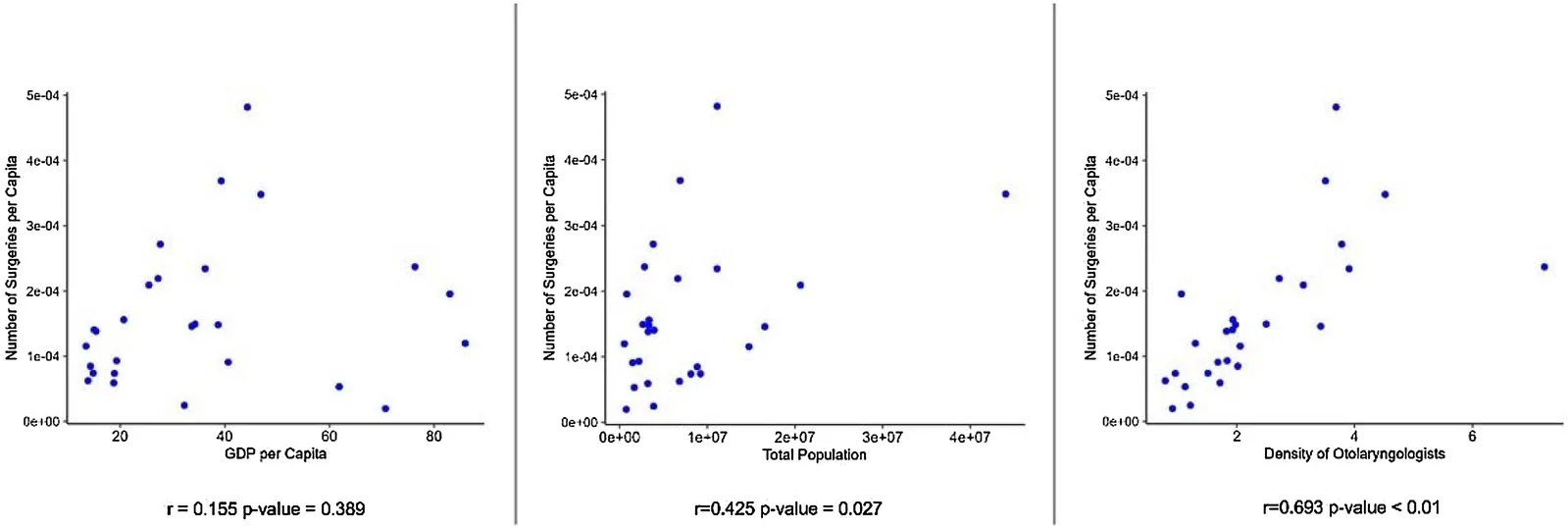
Correlation between otolaryngologic surgical rates and socioeconomic and workforce indicators. Panels show associations with GDP per capita, total population, and otolaryngologist density.

Multivariable analyses showed a significant association between otolaryngologist density and surgical rates (Fig. 3). Population size showed only a modest association, whereas GDP per capita was not significantly correlated with surgical provision. In the mixed-effects model predicting surgical rates per capita, otolaryngologist density remained the only significant positive predictor: each additional specialist per 100,000 inhabitants was associated with higher surgical output, whereas GDP per capita and population size showed no independent association. However, the second mixed-effects model indicated a diminishing marginal association of workforce expansion with surgical provision over time, with progressively smaller increases in surgical output corresponding to each additional specialist (Table 3).

**Table 3:** Mixed-Effects Model of Otolaryngologist Density and Surgical Provision in Brazil. Results of a mixed-effects regression model (lmer) estimating the association between otolaryngologist density and surgical rates per capita across Brazilian states. The model includes fixed effects for otolaryngologist density, GDP per capita, and year, with random intercepts by state. Coefficients (β) and p-values are reported, along with the estimated number of additional surgeries per 100,000 inhabitants associated with 1 additional otolaryngologist per 100,000 inhabitants. The intercept, otolaryngologist density in 2011, and GDP per capita represent baseline model effects. Annual rows show the estimated effect of otolaryngologist density for each year compared with 2011.

Effect	Coefficient (β)	p-value	Interpretation
Intercept	8.99E-06	0.762	Expected per capta surgeries in 2011, with otolaryngologist density = 0 and GDP = 0 (no direct practical meaning)
2011	1.16E-04	**<0.001**	Each additional otolaryngologist per 100,000 inhabitants associated with 11.6 surgeries per 100 000 inhabitants
GDP per capta	−1.53E-06	0.325	GDP was not significantly associated with per capita surgeries, after adjustment for other factors.
Effect of 1 additional otolaryngologist per 100,000 inhabitants, by year			
2013	−2.89E-05	**0.023**	≈ **8.7 surgeries per 100,000 inhabitants**
2015	−3.96E-05	**0.002**	≈ **7.6 surgeries per 100,000 inhabitants**
2018	−4.76E-05	**<0.001**	≈ **6.8 surgeries per 100,000 inhabitants**
2020	−7.42E-05	**<0.001**	≈ **4.2 surgeries per 100,000 inhabitants**
2024	−8.80E-05	**<0.001**	**≈ 2.8 surgeries per 100,000 inhabitants**

## Discussion

This study examines otolaryngologic surgical provision in Brazil between 2008 and 2024, focusing on the geographic distribution of specialists, surgical volumes, costs, and system-level factors associated with service delivery within the public health system. During the study period, the national density of otolaryngologists increased by 64.7%, while relative measures of inequality between states declined. Nevertheless, substantial regional disparities persisted, with higher workforce availability and surgical rates concentrated in the South and Southeast. Cluster analysis further indicated a socioeconomic gradient, with poorer regions showing lower workforce density, surgical rates, and expenditure levels than wealthier regions. Multivariable modelling indicated that otolaryngologist density was significantly associated with surgical rates per capita. However, the estimates presented in Table 3 suggest that this association weakened over time, which could represent progressively smaller gains in surgical output corresponding to each additional specialist. These findings align with the premise of the LCoGS that equitable surgical access in low-income and middle-income countries requires concurrent investments in workforce, infrastructure, and system capacity.^4^^,^^6^

The spatial heterogeneity observed in this study is consistent with patterns reported in other LMICs health systems, where specialists tend to be concentrated in economically developed regions.^3^^,^^7^ Although the coefficient of variation and Gini index for otolaryngologist density declined over time, substantial absolute differences between states remained. Persistently low densities in states such as Amapá, Roraima, and Acre could reflect ongoing challenges in attracting and retaining specialists in underserved regions. These disparities may originate early in the training pathway. According to the 2025 National Medical Demography Report, approximately 234 otolaryngology residency positions are offered annually in Brazil, with nearly 68% located in the Southeast and only about 3% in the North. This concentration of training opportunities could contribute to persistent workforce imbalances and may limit the renewal of specialists in regions with greater unmet surgical need.

The decline in surgical productivity per otolaryngologist observed during the study period could reflect multiple overlapping factors. The sharp reduction observed in 2020 coincided with the onset of the COVID-19 pandemic, a period characterized by widespread cancellation of elective procedures, reallocation of hospital resources, and restricted access to operating rooms. As a specialty that relies heavily on elective inpatient procedures, otolaryngology was particularly vulnerable to these disruptions.^12^ However, a gradual decline in the ratio of surgeries per specialist was already evident between 2011 and 2018. As shown in Table 1, this pattern occurred despite stable or increasing absolute surgical volumes and could partly reflect the rapid expansion of the otolaryngology workforce. Because surgical productivity was defined as the number of procedures performed per practicing specialist, a rapid increase in specialist density could mechanically reduce the number of surgeries attributed to each physician. These findings suggest that workforce expansion alone may not be sufficient to substantially increase surgical delivery without parallel investments in structural capacity. Limitations in operating room availability, anesthesia staffing, inpatient beds, and perioperative support could restrict the system’s capacity to absorb additional specialists. Similar patterns have been described across surgical specialties, suggesting that infrastructure and organizational capacity could represent important constraints on surgical productivity within public health systems.^13^

Approximately 70% of the surgical volume consisted of adenotonsillectomy, which reflects the routine surgical profile of otolaryngology within Brazil’s public health system, where this procedure is the most frequently performed operation. Nevertheless, the analysis was not limited to adenotonsillectomy alone and also encompassed other inpatient procedures with greater infrastructure requirements, including septoplasty, sinus surgery, tympanoplasty, and mastoidectomy, across which similar regional gradients were observed. Because the SIH/SUS database captures only inpatient procedures reimbursed within the public health system, the overall provision of otorhinolaryngologic care may be underestimated, particularly regarding outpatient procedures and services delivered in the private sector.

The “Now There Are Specialists” programme, launched by the Ministry of Health to expand priority specialties, represents a national initiative aimed at addressing workforce imbalances in specialist care. Otolaryngology was included among the programme’s priority areas, reflecting the specialty’s role in conditions affecting communication, airway function, and hearing. However, the findings of this study suggest that increases in the number of specialists alone may not necessarily translate into greater access to surgical care within the SUS. Although the otolaryngology workforce expanded during the study period, the number of surgeries performed per specialist declined, a pattern that could reflect limitations in structural capacity, including hospital infrastructure, operating room availability, anesthesia services, and perioperative support. These findings may provide useful considerations for the implementation of the programme, suggesting that workforce expansion could be more effective when accompanied by investments in hospital infrastructure and perioperative capacity at the regional level.^1^^,^^4^

From a policy perspective, these results suggest that workforce expansion and infrastructure development may need to progress in parallel to support improvements in surgical provision. Expanding the number of specialists without corresponding structural capacity could potentially result in underutilized workforce capacity and persistent geographic disparities in access to care. The diminishing marginal associations observed in this study could also reflect constraints in existing surgical capacity in some regions, where additional specialists may not translate into higher surgical output in the absence of investments in hospital beds, equipment, and support staff.^4^, ^5^, ^6^, ^7^ Similar patterns have been described in other middle-income countries, where workforce growth occurred alongside more limited expansion of surgical infrastructure.^14^

In a global context, Brazil’s otolaryngology workforce density remains relatively modest. In 2024, the country reached 3.97 otolaryngologists per 100.000 inhabitants, less than half the levels observed in high-income countries such as Germany or France, where densities exceed 7.0 per 100.000 and distribution is more balanced.^13^^,^^15^^,^^16^ In contrast, Brazil’s regional patterns appear more comparable to those observed in other middle-income Latin American countries. In countries such as Colombia and Chile, surgical and otolaryngologic services are concentrated in large metropolitan centers, while rural and peripheral regions often face persistent shortages of specialists and longer waiting times for elective procedures.^17^, ^18^, ^19^ Similar to Brazil’s SUS, these systems operate under decentralized health frameworks with marked regional inequalities in access to specialized surgical services. To our knowledge, this study is among the first national-level analyses to examine the diminishing marginal association between specialist expansion and surgical provision within a public health system.

In comparison with international data, the proportion of otolaryngologic surgeries observed in Brazil appears lower than that reported in high-income countries. National surgical rates in our study ranged approximately from 8 to 34 otolaryngologic procedures per 100,000 inhabitants, depending on the state cluster, with a national average close to 20 procedures per 100,000 inhabitants, despite the observed increase in specialist density over time. In contrast, population-based data from the United States report otolaryngologic surgical rates commonly exceeding 40–50 procedures per 100,000 inhabitants per year.^1^^,^^19^ Similarly, data from Western Europe describe otolaryngologic surgical rates ranging from 30 to 50 procedures per 100,000 inhabitants. These comparisons suggest that higher surgical rates may be more likely when workforce expansion occurs alongside broader growth in health system capacity.^20^

In middle-income and lower-resource settings, reported otolaryngologic surgical rates are generally lower, often below 10–20 procedures per 100,000 inhabitants, even in countries with expanding specialist workforces. International assessments suggest that, in these contexts, increases in specialist numbers do not always translate into proportional growth in surgical provision.^21^ The Brazilian experience appears consistent with this pattern. Although the otolaryngology workforce expanded substantially during the study period, the number of surgeries performed per specialist declined over time. A further reduction was observed in 2020, coinciding with the onset of the COVID-19 pandemic. From an international perspective, Brazil may therefore occupy an intermediate position between high-income systems with higher surgical throughput and lower-resource settings where surgical provision remains more limited. These observations suggest that expanding specialist numbers alone may not necessarily lead to proportional increases in surgical delivery within the public health system.^12^

The cost analysis presented in Table 3 showed persistent regional differences in public expenditure per otolaryngologic procedure, with the South and Southeast reporting the highest average values. For complex procedures such as advanced otologic surgeries, average expenditures in the Southeast were more than twice those observed in the North. These differences could be associated with structural factors, as wealthier regions may have greater availability of specialized surgical centres, broader access to high-cost consumables and technology, and a higher proportion of complex procedures.^22^, ^23^, ^24^ In contrast, poorer regions may present a case mix more concentrated in lower-cost routine procedures, which could be related to differences in available equipment and subspecialty expertise.^25^, ^26^, ^27^

For policymakers implementing the “Now There Are Specialists” initiative, these findings may offer considerations regarding the broader conditions required to support improvements in surgical provision. Expansion of training programmes and hiring quotas could be more effective when accompanied by efforts to strengthen the physical and operational capacity of the SUS and referral coordination across levels of care. Sustainable implementation may also depend on stable financing mechanisms for surgical services at the federal and state levels, as well as policies that encourage the retention of specialists in underserved regions through structured career pathways, targeted funding, and regional infrastructure development. Without such coordination, increases in the number of specialists alone may not necessarily translate into substantial improvements in access, particularly for populations who rely primarily on the public health system.

Finally, these findings reinforce the need for Brazil’s ongoing health reforms to adopt a system-level approach to surgical care. Aligning workforce planning with infrastructure investment, financing, and equitable regional distribution can transform the “Now There Are Specialists” programme into a long-term instrument for reducing surgical inequities. Ultimately, the programme has the potential to reshape surgical equity in Brazil if implemented as part of an integrated system-strengthening agenda, one that treats workforce expansion not as an end, but as a catalyst for broader investment in hospitals, infrastructure, and perioperative care. The evidence presented here provides policymakers with concrete data to guide this alignment and to ensure that the expansion of otolaryngology training and practice translates into tangible improvements in surgical access, outcomes, and equity across Brazil.

### Limitations

This study has limitations. It relies on secondary data from SIH/SUS, which, despite nationwide coverage and mandatory reporting, may contain underreporting or miscoding, particularly in remote areas. Data are restricted to the public system, excluding private-sector provision that is substantial in wealthier regions. In addition, the database does not include patient-level variables or detailed facility-level information, such as operating room availability or anesthesia capacity. Nonetheless, its comprehensiveness and standardized structure make it a valuable source for national analyses of surgical provision. Furthermore, given the ecological, state-level design of this study, the associations observed should be interpreted with caution and cannot be considered evidence of causal relationships. Although infrastructure constraints represent a plausible explanation for the declining marginal effect of workforce density on surgical output, other unmeasured factors, including organizational efficiency, referral dynamics, case-mix complexity, and regional policy variation, may also contribute to these patterns.

## Conclusion

The expansion of the otolaryngology workforce in Brazil was accompanied by comparatively smaller increases in surgical provision across regions. Despite substantial growth in specialist numbers, surgical delivery appears to remain limited. These findings suggest that improvements in surgical access may also depend on broader health system capacity.

## ORCID ID

Arthur Menino Castilho: 0000-0002-9024-8004

## Authors’ contributions

LSS and AMC contributed equally to the conceptualization and design of the study, data collection, formal analysis, data interpretation, original manuscript drafting, critical revision of important intellectual content, and approval of the final manuscript version. SFO contributed to data analysis, result interpretation, original manuscript drafting, manuscript revision, and critical revision of important intellectual content. RGFA contributed to data collection, manuscript revision, assistance in data interpretation, and critical revision of important intellectual content. VARS contributed to data analysis, manuscript revision, and critical revision of important intellectual content. All authors approved the final version of the manuscript and agree to be accountable for all aspects of the work.

## Funding

No financial or material support was received for this study.

## Data availability statement

The authors declare that all data are available in repository.

## Data availability

The data that has been used is confidential.

Data will be made available on request.

## Declaration of competing interest

The authors declare no conflicts of interest.

## References

[1] Petrucci A.G., Mello A.L.S., Lira A.L.B.C. (2023). Global health and otolaryngology: needs, priorities, and future directions. JAMA Otolaryngol Head Neck Surg..

[2] Bhutta M.F., Bu X., Dewan K. (2019). WHO World Hearing Report: implications for global Hearing care. BMJ Open..

[3] Scheffer M., Cassenote A., Guerra A. (2025).

[4] Meara J.G., Leather A.J.M., Hagander L. (2015). Global surgery 2030: evidence and solutions for achieving health, welfare, and economic development. Lancet..

[5] Rose J., Weiser T.G., Hider P., Wilson L., Gruen R.L., Bickler S.W. (2015). Estimated need for surgery worldwide based on prevalence of diseases: a modelling strategy for the WHO Global Health Estimate. Lancet Glob Health..

[6] Bath M., Bashford T., Fitzgerald J.E. (2019). What is global surgery? Defining the multidisciplinary interface between surgery, anesthesia, and public health. BMJ Glob Health..

[7] Dubowitz G., Detlefs S., McQueen K.A.K. (2010). Global anesthesia workforce crisis: a preliminary survey revealing shortages contributing to undesirable outcomes and unsafe practices. World J Surg..

[8] Notrica M.R., Evans F.M., Knowlton L.M., McQueen K.A.K. (2016). Rationale for developing a regional trauma network in Latin America. World J Surg..

[9] Poenaru D., Pemberton J., Frankfurter C., Cameron B.H., Alvarez M.P., Ozgediz D. (2014). Establishing a baseline for global pediatric surgical capacity in Guatemala. World J Surg..

[10] Ministério da Saúde (Brasil). Departamento de Informática do SUS (DATASUS). Sistema de Informações Hospitalares do SUS – SIH/SUS.

[11] Instituto Brasileiro de Geografia e Estatística (IBGE). Instituto Brasileiro de Geografia e Estatística. Available from: https://www.ibge.gov.br. Accessed August 19, 2025.

[12] Nepogodiev D, Bhangu A; COVIDSurg Collaborative (2020). Elective surgery cancellations due to the COVID-19 pandemic: global predictive modelling to inform surgical recovery plans. Br J Surg..

[13] Massenburg B.B., Jenny H.E., Saluja S. (2017). Barriers to surgical care in low- and middle-income countries: a systematic review. Bull World Health Organ..

[14] Gupta S., Reddy C.L., Meara J.G. (2020). Surgical workforce capacity and infrastructure in low-income countries: a global surgery perspective. World J Surg..

[15] Alkire B.C., Raykar N.P., Shrime M.G. (2015). Global access to surgical care: a modelling study. Lancet Glob Health..

[16] Farmer P.E., Kim J.Y. (2008). Surgery and global health: a view from beyond the OR. World J Surg..

[17] Holmer H., Bekele A., Hagander L. (2019). Evaluating the collection, comparability, and findings of six global surgery indicators. Br J Surg..

[18] Shrime M.G., Dare A.J., Alkire B.C., Meara J.G. (2016). A global country-level comparison of the financial burden of surgery. Br J Surg..

[19] Saraswathula A., Gourin C.G., Stewart C.M. (2021). National trends in US otolaryngology surgical volume during the early COVID-19 pandemic. JAMA Otolaryngol Head Neck Surg..

[20] Rimmer J., Hellings P., Lund V.J. (2019). European position paper on diagnostic tools in rhinology. Rhinology..

[21] Hathi K., Nam Y.S.J., Fowler J. (2024). Improving operating room efficiency in otolaryngology-head and neck surgery: a scoping review. Otolaryngol Head Neck Surg..

[22] Pan American Health Organization (PAHO) (2021).

[23] Ribeiro A.L.P., Duncan B.B., Brant L.C.C. (2020). Health system inequalities and regional variation in mortality in Brazil: a national longitudinal study. Lancet..

[24] Shrime M.G., Sleemi A., Dare A.J. (2015). Catastrophic expenditure to pay for surgery worldwide: a modelling study. Lancet Glob Health..

[25] Massenburg B.B., Saluja S., Jenny H.E. (2019). Assessing the Brazilian surgical system with six global surgery indicators: a population-based study. World J Surg..

[26] Biccard B.M., Madiba T.E., Kluyts H.L. (2018). Perioperative patient Outcomes in the African Surgical Outcomes Study: a 7-day prospective observational cohort Study. Lancet.

[27] Weiser T.G., Haynes A.B., Molina G. (2015). Global surgical volume: an estimation from available data. Lancet..

